# Molecular Detection of *Ehrlichia chaffeensis* in *Amblyomma parvum* Ticks, Argentina

**DOI:** 10.3201/eid1412.080781

**Published:** 2008-12

**Authors:** Laura Tomassone, Pablo Nuñez, Ricardo E. Gürtler, Leonardo A. Ceballos, Marcela M. Orozco, Uriel D. Kitron, Marisa Farber

**Affiliations:** Università di Torino, Torino, Italy (L. Tomassone); Instituto de Biotecnología, INTA, Castelar, Argentina (P. Nuñez, M. Farber); Universidad de Buenos Aires, Buenos Aires, Argentina (R.E. Gürtler, L.A. Ceballos, M.M. Orozco); Emory University, Atlanta, Georgia, USA (U.D. Kitron)

**Keywords:** Ehrlichia chaffeensis, Ixodid ticks, Amblyomma parvum, HGE, northern Argentina, letter

**To the Editor:** *Ehrlichia chaffeensis* is an obligate intracellular bacterium in the family *Anaplasmataceae*. It is considered an emerging pathogen in the United States because it is the causative agent of human monocytotropic ehrlichiosis (*1*), a flu-like illness that can progress to severe multisystem disease and has a 2.7% case-fatality rate (*2*).

In Central and South America, human cases of ehrlichiosis with compatible serologic evidence have been reported in Venezuela, Brazil, Mexico, and Chile, although the bacterium has not been isolated (*3*). Recently, molecular evidence of *E. chaffeensis* infection was reported for a symptomatic 9-year-old child in Venezuela (*4*). In Argentina, antibodies reactive to *E. chaffeensis*, or an antigenically related *Ehrlichia* species, were detected in human serum samples during a serologic survey in Jujuy Province, where fatal cases of febrile illness were reported (*5*).

During November–December 2006, we collected ticks by dragging the vegetation and by examining mammal hosts, including humans, in semiarid southern Chaco, Argentina, Moreno Department, Province of Santiago del Estero. Ticks, kept in 70% alcohol, were identified as *Amblyomma parvum* (n = 200), *A. tigrinum* (n = 26), and *A. pseudoconcolor* (n = 13). A sample of 70 *A. parvum* and 1 *A. tigrinum* ticks collected on domestic ruminants and canids were subjected to PCR and reverse line blot hybridization by using the TBD-RLB membrane (Isogen Life Science, Maarssen, the Netherlands) (*6*) to look for *Anaplasma* and *Ehrlichia* spp. DNA was extracted from individual ticks by using the DNeasy Blood and Tissue kit (QIAGEN Valencia, CA, USA); several negative controls (distilled water) for both DNA extraction and PCRs were run alongside the samples in random order throughout the experiments. Primers Ehr-R (5′-CGGGATCCCCAGTTTGCCGGGACTTYTTCt-3′) (*6*) and Ehr-Fint (5′-GGCTCAGAACGAACGCTG-3′; Inst. Biotecnologia, Instituto Nacional de Tecnología Agropecuaria, unpub. data) were used to amplify a 500-bp fragment of the 16S gene of *Anaplasma/Ehrlichia* spp. PCR products were analyzed by reverse line blot hybridization, and 11.3% (95% confidence interval [CI] = 4.9–21.0) showed a positive signal to the specific *E. chaffeensis* probe: 8 *A. parvum* ticks collected from a dog (n = 1), a fox (*Lycalopex gymnocercus*, n = 1), goats (n = 2), and cattle (n = 4). No signals to other probes present in the membrane were recorded (*A. phagocytophylum*, *A. marginale*, *A. centrale*, *A. ovis*, *E. ruminatium*, *E.* sp. *Omatjenne*, *E. canis*). Further sequence analysis of 16S fragments confirmed the result, with our sequences showing 99.6% identity with the corresponding fragment of the *E. chaffeensis* strain Arkansas 16S gene (GenBank accession no. EU826516). To better characterize the positives samples, we then amplified variable-length PCR target (VLPT) of *E. chaffeensis* (*7*). PCR products of variable length were detected by conventional gel electrophoresis analysis (Figure). Distilled water and *R. conorii* DNA were used as negative controls, and *E. chaffeensis* DNA as the positive control. The finding was confirmed by sequence analysis (GenBank accession nos. EU826517 and EU826518)

**Figure:**
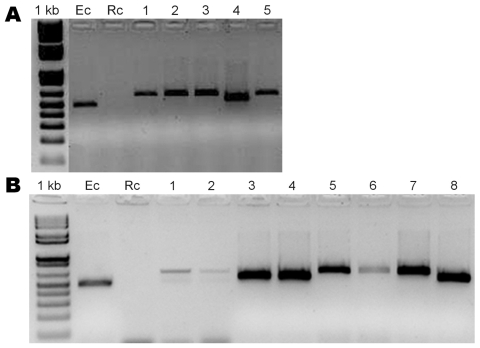
Agarose gel electrophoresis of PCR products amplified with *Ehrlichia chaffeensis* (Ec) variable-length PCR target primers. Rc, *Rickettsia conorii* (negative control). The sources of DNA templates used for amplification are *Amblyomma parvum* ticks collected from different hosts: A) 1–5 humans; B) 1 dog, 2 foxes, 3–6 cattle, 7–8 goats. Variable amplicon size represents different genotypes that result from differences in the number of tandem repeats in the 5′ end of the variable-length PCR target; PCR products’ sizes range from 500 bp to 600 bp.

In view of these positive results, another set of 108 specimens was tested by *E. chaffeensis* VLPT PCR: all the ticks collected on humans (80 *A. parvum*, 1 *A. pseudoconcolor,* and 4 *A. tigrinum*), 18 host-seeking *A. parvum* ticks, and 5 *A. parvum* ticks collected on armadillos of the genera *Tolypeutes* and *Chaetophractus*. *E. chaffeensis* was detected in *A. parvum* ticks only: 5 from humans (6.2%; 95% CI 2.1–14.0; Figure, panel A) and 3 fromhost-seeking ticks (16.7%; 95% CI 3.6–41.4). In total, *E. chaffeensis* was detected in 9.2% (95% CI 5.4–14.6) of tested *A. parvum* ticks in the study area. Of the 16 positive *A. parvum*, 5 were infesting humans.

Little is known about *E. chaffeensis* epidemiology in South America. In Brazil, wild marsh deer (*Blastocerus dichotomus*) are suspected to be its natural reservoir, but the tick involved in the transmission cycle is not known (*8*). In North America, *E. chaffeensis* sp. is maintained principally by the lone-star tick, *A. americanum,* and the white-tailed deer (*Odocoileus virginianus*) (*2*). However, the possibility of transmission by different ticks and infection among other hosts has been reported; specific antibodies to *E. chaffeensis* were detected in domestic and wild canids and goats (*2*), and recently experimental infection was demonstrated in cattle (*9*). We did find *E. chaffeensis* organisms in ticks collected on both wild and domestic animals, but the possible role of different mammals as reservoir hosts deserves further investigation. Moreover, the finding of polymorphic VLPT gene fragments in our sample indicates the circulation of *E. chaffeensis* genetic variants in the study area. VLPT repetitive sequences vary among isolates (*7*); however, it is not known whether genetic variants differ in pathogenicity or are correlated with geographic distribution or host range.

All positive ticks were *A. parvum*, a common tick of domestic animals that frequently feeds on humans in Argentina and Brazil and is considered a potential vector of zoonoses (*10*). In our study area, this tick species was by far the most abundant on humans (93.2%), and our results suggest its potential role as a vector of *E. chaffeensis*.
